# Maternal Depression and Associated Factors Among Pregnant Women Attending Ante Natal Care, Southern Ethiopia: Cross-Sectional Study

**DOI:** 10.3389/fpubh.2022.848909

**Published:** 2022-06-09

**Authors:** Yacob Abraham Borie, Melese Siyoum, Aklile Tsega, Gemechu Anbese

**Affiliations:** Faculty of Health Sciences, College of Medicine and Health Sciences, Hawassa University, Hawassa, Ethiopia

**Keywords:** maternal depression during antenatal care, Southern Ethiopia maternal depression, antenatal care, pregnant, women

## Abstract

**Background:**

Patients with depressed mental disorders may experience a lack of interest or pleasure, a poor mood, feelings of guilt or unworthiness, sleep and appetite disturbances, and easy fatigability. Based on the degree of the condition, depression is classed as mild, moderate, or severe. Maternal depression is the most common psychiatric condition during pregnancy, and its harmful effects have serious ramifications for both the mother and the fetus. Almost one in every four women will experience depression at some point in her life, the majority of which will occur during her childbearing years. Studies reports showed that antenatal depression is a common maternal problem in Ethiopia and as reported antenatal depression ranges in Ethiopia from 19.04 to 29.92%.

**Objective:**

To assess the prevalence of maternal depression and associated factors among antenatal care attendants at Wolayta Sodo Teaching and Referral hospital, Southern Ethiopia.

**Methods:**

Health facility based cross-sectional study was conducted at Wolayta Sodo Teaching and Referral Hospital from May 01 to 30, 2018. Data were collected from through face to face interview at exit from antenatal care unit using structured questionnaire and checklist adopted from patient Health Questionnaire (PHQ-9). Data were collected from 309 antenatal attendant mothers using systematic random sampling from each either mother. Binary and multivariable logistic regression model was employed to identify factors associated with maternal depression at *P*-value <0.05 level of significant.

**Result:**

Depression among pregnant mother was found to be 27.2% (95% Cl: 22, 32%). Women's level of education; being elementary school (AOR = 6.35 95%CL (2.32, 17.38), completing high school and above (AOR = 3.35, 95%CL 1.33, 8.47) were associated with maternal depression whereas having good husband support was protective for maternal depression (AOR = 0.4, 95%CL: 0.19, 0.83) and also not using substance during pregnancy period was protective for maternal depression (AOR = 0.39, 95%CI, 0.19, 0.77).

**Conclusion:**

The frequency of mother depression in this community was greater than in previous Ethiopian studies reported. Maternal depression was linked to a woman's level of education, husband support, and substance usage. This suggested that health care providers regarding pregnant women should emphasize the importance of husband support, substance usage and women level of education.

## Introduction

Patients with depressed mental disorders may experience a lack of interest or pleasure, a poor mood, feelings of guilt or unworthiness, sleep and appetite disturbances, and easy fatigability. Based on the degree of the condition, depression is classed as mild, moderate, or severe (1). It is the most common psychiatric condition during pregnancy, and its harmful effects have serious ramifications for both the mother and the fetus. Almost one in every four women will experience depression at some point in her life, the majority of which will occur during her childbearing years. Study done in northern Ethiopia reported the prevalence of antenatal depression 31.1% (1, 2). Systematic review done in Ethiopia showed that antenatal depression in Ethiopia ranges from 19.04 to 29.92% and it reported that antenatal depression is a common maternal problem in Ethiopia (3). Low birth weight is linked to maternal depression during pregnancy, which is a major public health issue in underdeveloped nations (4). Antenatal depression is predicted to affect 15.6 percent of women in low- and middle-income nations (5).

Evidence suggests that depression has an influence on child health in poor and middle income nations that goes beyond psychosocial developmental delays (6). Pregnant women who were depressed were more likely to have obstetrical complications such as pre-eclampsia, uterine irritability, and pregnancy-induced hypertension (7).

Even in well-resourced frameworks, depression during the prenatal period was often not acknowledged, and treatment rates in pregnant women were lower and slower than in non-pregnant women (8). It also has an impact on the mother-infant relationship by influencing the occurrence of post-natal depression (9).

Despite the fact that depression has a significant impact on pregnant mothers, according to our literature review, research in this area is limited. This study was conducted with the goal of determining the prevalence and factors related with depression among Southern Ethiopian pregnant women attending antenatal care.

## Methods and Materials

### Study Area and Period

The study was conducted at Wolata Sodo University teaching and referral Hospital from May 1–30, 2018. The hospital is located in Sodo town, which is found 327 km far from Addis Ababa to southern direction. The hospital is expected to serve around two million people. The total number of bed in the hospital is 200, out of which 60 beds were in obstetrics and gynecology department. The services delivered by these departments were pre-operative, post-operative, in patient, post abortion, safe abortion service, labor, delivery, ANC service and others. Currently 257 health professionals serve permanently in hospital including specialist, general practitioners nurses, midwives, pharmacists and others. The average number of monthly ante natal care service was around 681.

### Study Population

The study population was all pregnant women who came to attend ANC unit during the study period at Wolayta Sodo University teaching and referral hospital.

#### Inclusion Criteria

Pregnant women who came to attend for ANC at Wolayta Sodo Univesity teaching and referral hospital.

#### Exclusion Criteria

Pregnant mothers who were suffering from pain and bleeding were excluded.

### Sample Size and Sampling Technique

Sample size was determined using single population proportion formula with the assumption of 24.9%, proportion of maternal depression (7). Confidence level of 95% confidence level and marginal error of 5%. After adding 10% for non-response rate, the final calculated sample size was 313. Participants were included systematically when they enter for ante natal care.The number of client flow in the last 1 month prior to the data collection period was used to calculate the k^th^ value which was two. Accordingly, each woman was included in the study when receive care at antenatal care unit.

#### Data Quality Control Issues

Ensured by conducting the pretest among 5% sample. Training was given for the data collectors on the data collection tool and sampling techniques and procedures. Supervision was held regularly and necessary feedback was offered to data collectors.

### Data Collection

The data were collected by five trained psychiatry nurses through face to face interview using structured questionnaires on paper. The data collection equipment was made up of different parts

The first part was socio-demographic information, second part included obstetric history of pregnant women husband support for pregnant women and substance use of women during the pregnancy period finally maternal depression was measured using standardized checklist adopted from patient Health Questionnaire (PHQ-9).

PHQ-9 is composed of nine questions, each with four possible responses. Each response was assigned a score ranging from zero to three to indicate the severity of symptom. The overall value of a scale ranges from 0–27 and scored as mild depression when the total scores is 5 to 9, moderate depression when the total scores is 10 to 14,moderately severe depression when the total scores is 14 to 19 and severe depression accounts >20.

In our study we defined depression from mild to severe one based on the scale. So PHQ-9 scale cut pint five to nine taken to define depression among antenatal women.

### Data Processing and Analyses

For analysis, all completed surveys were loaded into Epi-Data version 3.1 and exported to SPSS version 22. The study participants were described using descriptive analysis based on their basic background characteristics, and maternal depression among ANC-attendants was estimated using descriptive analysis. To choose independent variables for inclusion in multivariable logistic regression, binary logistic regression was employed. In the bivartte analysis, variables with *p*-values ≤ 0.25 were entered into the multivariable logistic regression model. Multivariable logistic regression was utilized to find the factors of mother depression among ANC attendant women. The presence and intensity of association were assessed using adjusted odds ratios (AORs) with 95% confidence intervals (CIs) derived from multivariable logistic regression.

## Result

### Socio Demographic History

From 313 pregnant women only four pregnant women were eliminated from the study because they dropped out during the interview due to physical discomfort associated with pregnancy, leaving 309 pregnant women with gestational ages ranging from 16 to 39 weeks and a 98.7% response rate. Women ranged in age from 15 to 38, with a mean and standard deviation of 25.47 and 5.58, respectively. In number 185 or 59.9% of the women in this study were between the ages of 20 and 29, and in terms of residence 215 (69.6%) were from the urban, in terms of religion; 190 (61.5%) were protestants, and marital status showed that 271 (87.7%) were married (see Table 1).

**Table 1:** Socio demographic characteristics of pregnant women who were at antenatal care service and participated in study at Wolayta Sodo teaching and referral Hospital, June, 2018.

**Variable**	**Category**	**Number**	**Percent**
Age	14–19	32	10.1
	20–29	185	59.9
	>30	92	29.8
Residence	Urban	215	69.6
	Rural	94	30.4
Religion	Orthodox	78	25.24
	Catholic	23	7.4
	Protestant	190	61.5
	Muslim	14	4.5
	Others (specify)	4	1.3
Marital status	Single	12	3.9
	Married	271	87.7
	Divorce	15	4.9
	Widowed	11	3.6
Ethnicity	Oromo	20	6.5
	Sidama Amhara	2,135	6.8 11.3
	Wolayta	233	75.4
Education	Not attend formal educated	53	17.2
	Elementary school (from grade 4 to 8)	71	23.0
	Completed high school and above	185	59.9
Occupation	Farmer	41	13.3
	Merchant	55	17.8
	Government employee	78	25.2
	House wife	135	43.7
Family income (in ETB)	1. <1000	118	38.2
	2.1001–2000	69	22.3
	3.2000–3000	54	17.5
	4. >3000	68	22.0

### Obstetric History of Pregnant Mothers

In this study 101 (32.7) of them were primi gravida while 50 (16.4) of multigravida mothers had at least one time history of abortion and 213 (68.7) of the pregnancy was planned and wanted (see Table 2).

**Table 2:** Obstetric characteristics of pregnant women who attended ANC service and participated in the study during study period at Wolayta Sodo teaching and referral Hospital, June 2018 (created by authors).

**Variables**	**Categories**	**Number**	**(%)**
Number of previous pregnancy (Gravidity)	No	101	32.7
	One	80	25.9
	Two	60	19.4
	Three	35	11.3
	Four and above	33	10.6
Number of previous abortion	No	251	81.2
	One	50	16.2
	Two	6	1.9
	Three and above	2	0.6
Number of children <5 years old	No One	175 106	56.6 34.3
	Two and above	28	9.1
History of previous pregnancy complication	No	254	82.2
	Yes	55	17.8
History of previous depression	No	305	98.7
	Yes	4	1.3
Medical illness	No	249	80.6
	Yes	60	19.4
The pregnancy wanted and planned	Planned	213	68.9
	Unplanned	96	31.1
Husband support	Good	269	87
	Not good	40	13
Substance	No	259	83.8
	Yes	50	16.2

### Prevalence of Depression in Pregnant Mothers

The nine items of the Patient Health Questionnaire (PHQ-9) were added together to create a single variable depression. The new variable's absolute value ranges from 0 to 27. In this study, the overall prevalence of depression was 27.2 percent (95 percent CI: 22.0, 32.0). A total of 47 (15.2%) women had mild depression (PHQ-9 score of 5 to 9) and 27 (8.73%) experienced moderate depression during pregnancy (PHQ-9 score of 10 to 14), 10 (3.23%) had severely moderate depression (PHQ-9 score 14 to 19) but 0 severe depression based on PHQ-9 tool. So overall number of women with depression was 84 in number and 27.2% in percentage.

### Factor Associated With Maternal Depression

Women's education, substance use, and husband support were found to have *P*-values <0.25 in bi-variable logistic regression and were included for multivariable logistic regression models using binary logistic regression. In multivariable logistic regressions, all three variables were significantly associated with maternal depression: women's level of education (AOR = 3.35, 95% CI: 1.32, 8.47), husband support (AOR = 0.40, 95% CI: 0.19, 0.83), and substance use (AOR = 0.39, 95% CI 0.19, 0.77) (see Table 3).

**Table 3:** Factors associated with depression among pregnant women who attended antenatal care service and participated in the study during study period at Wolayta Sodo teaching and referral Hospital, 2018 (created by authors).

**Variables**	**Categories**	**Depression**		**COR**	**AOR (95% CI)**	***P*** **-value**
		**No**	**Yes**			
*Level of education	No formal education	47	6	1	1	
	Elementary	43	28	5.101	6.353 (2.32,17.38)	0.001
	Completed high school and above	135	50	2.901	3.350 (1.32,8.47)	0.00
*Husband support	Good	203	66	0.397	0.402 (0.19, 0.83)	0.014
	Not good	22	18	1	1	
*Substance use	No	195	64	0.492	0.39 (0.19, 0.77)	0.007
	Yes	30	20	1	1	

* *Shows significant association at p-value <5%*.

## Discussion

Depression was found to be present in 27.2 percent of antenatal care attendant women in the current study (95 percent CI: 22.0, 32.0). This outcome is consistent with findings from a study of low-income African American and Caucasian women (27 %) (10), in South Africa (21–47%) (11), in Addis Ababa, Ethiopia (24.9%) (7), Gondar University hospital (23%) (12).

This study prevalent was lower than study done in rural South Africa (47%) (13). This may be difference in study population, in this study most of pregnant women were from urban area and the study report from South Africa showed pregnant women from rural area. Living in rural area has more burden than urban area since there is difficulty of access and affordability for necessary services.

This study prevalence was higher than from systematic review report of perinatal depressive disorder prevalence in Africa (14). The possible explanation could be difference tools used.

In this study, the odds of developing maternal depression were more than three times higher among women who had completed high school or higher than women who had no formal education. A study conducted in Sweden backs this up (15) as well as a research conducted in the United States (16). This might be women completed high school and above could have additional responsibilities particularly related with job and had limited time for caring themselves than women with no formal education.

Maternal depression was 60% less likely in women who had good husband support than in those who had no good husband support. This was supported by study which revealed that husband support during pregnancy had positive effect on maternal health (17). This can be explained by the fact that husband support makes women feel psychologically solid and useful and also good husband support is indicative of strong social support.

When compared to women who use substances, pregnant women who do not use substances were 51% less likely to suffer maternal depression than pregnant women who use substances. A study conducted in Mizan Aman town, Bench Maji, Southwest Ethiopia, backs up this claim (3). This was expected as known that substance use during even before pregnancy has adverse effect on mental wellbeing of women. Furthermore this report indicated that 16.2% of women were using substances during the current pregnancy this in turn does not only affect mental wellbeing of pregnant women but also could have negative adverse effect on physical and mental health of both pregnant women and fetus. Adverse effect of physical and mental health of pregnant women and also outcome of the fetus.

## Conclusion

The frequency of mother depression in this community was greater than in previous Ethiopian studies reported. Maternal depression was linked to a woman's level of education, husband support, and substance usage.

## Data Availability Statement

The original contributions presented in the study are included in the article/supplementary material, further inquiries can be directed to the corresponding author.

## Ethics Statement

Ethical clearance was obtained from Ethical Review Board of the Hawassa University.

## Author Contributions

All authors conceived the study and were involved in the study design, reviewed the article, analysis, report writing, and drafted the manuscript. All authors contributed to the article and approved the submitted version.

## Conflict of Interest

The authors declare that the research was conducted in the absence of any commercial or financial relationships that could be construed as a potential conflict of interest. The reviewer TF declared a shared affiliation with the authors to the handling editor at time of review.

## Publisher's Note

All claims expressed in this article are solely those of the authors and do not necessarily represent those of their affiliated organizations, or those of the publisher, the editors and the reviewers. Any product that may be evaluated in this article, or claim that may be made by its manufacturer, is not guaranteed or endorsed by the publisher.

## Abbreviations

ANC: Antenatal care
BDI: Back depression inventory
CIDI: Composite international diagnostic interview
CMD: common mental disorder
MCH: Maternal and child health
PHQ-9: Patient health questionnaire
SNNPRS: South nation nationality and peoples of regional state.
